# Successful treatment of scapholunate advanced collapse: A case report

**DOI:** 10.1002/ccr3.2201

**Published:** 2019-05-14

**Authors:** Sunny R. Kim

**Affiliations:** ^1^ Progressive Rehabilitation Medicine, PC Cedar Rapids Iowa

**Keywords:** amniotic tissue, avascular necrosis, osteoarthritis, scapholunate dissociation, umbilical cord, wrist

## Abstract

This case illustrates the successful treatment by injection of amniotic membrane and umbilical cord particulate for scapholunate advanced collapse unresponsive to traditional nonsurgical treatment.

## 1 INTRODUCTION

Osteoarthritis (OA) of the wrist is one of the most common conditions encountered by hand surgeons and is characterized by degeneration of cartilage and hypertrophic bone changes.^1^ These abnormalities result in altered wrist kinematics, which can lead to debilitating pain, swelling, and loss of motion as the involved joints degenerate. Factors such as increasing age, female gender, obesity, and genetics are also believed to contribute to the development of hand or wrist OA.^2^ Scapholunate advanced collapse (SLAC) is a frequently encountered form of wrist OA that typically ensues from scapholunate ligament injury.^3^ In fact, SLAC is the most common etiology of wrist OA, accounting for approximately 55% of all individuals with wrist arthritis.^4^

Conservative treatment for alleviating wrist OA pain includes splint immobilization, nonsteroidal anti‐inflammatory drugs (NSAIDs), and intra‐articular corticosteroid injections.^1^ However, these treatments tend to provide temporary pain relief.^5^ In addition, the cardiovascular, gastrointestinal, and renal toxicities of NSAIDs have limited their long‐term use, particularly in the elderly.^6^ Thus, there is a need for a safe, conservative OA treatment that alleviates pain, improves function, and slows joint degeneration. One potential solution is cryopreserved amniotic and umbilical cord (AMUC) tissue, which has been used in a variety of clinical applications due to their anti‐inflammatory and proregenerative properties.^7^, ^8^, ^9^, ^10^, ^11^ AMUC is known to upregulate anti‐inflammatory mediators and promote apoptosis of pro‐inflammatory cells that can lead to succession of pain.^7^ Recently, injection of AMUC particulate matrix has been shown to attenuate progression of knee OA in preclinical and clinical studies.^12^, ^13^ Herein, we report the safe use and effectiveness of AMUC injection in a case of wrist OA associated with SLAC that had failed prior conservative treatment.

## 2 CASE PRESENTATION

**Figure 1:**
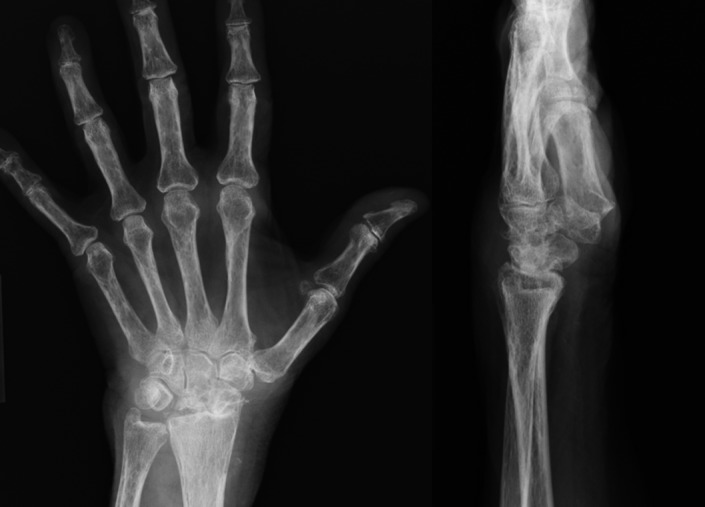
Case presentation. Patient had left wrist osteoarthritis with scapholunate dissociation and avascular necrosis of the lunate

The patient is an 85‐year‐old Caucasian female (5′1″, 30.2 kg/m^2^ BMI) who presented with proximal, distal, dorsal, and ventral pain of her left wrist. Her medical history was notable for type 2 diabetes, hypercholesterolemia, and hypertension. The aching, dull and stiff pain in her left wrist gradually started one year prior. Diagnostic X‐ray examination showed severe degenerative joint disease of the left wrist with scapholunate dissociation, scapholunate ligament tear, and avascular necrosis of the lunate (Figure 1). The patient was subsequently treated with corticosteroid injection of the wrist which only provided relief for less than a week. Other treatments including home care and over‐the‐counter pain medication did not provide relief. Due to the recalcitrant nature, the attending physician recommended fusion or wrist replacement, which the patient declined and sought for a second opinion.

At presentation, her pain score was an average of 6 out of 10, but periodically reached 10 out of 10 on a numerical rating scale (0 being no pain, 10 being worst possible pain). The pain was reportedly constant but worsened with gripping, twisting, and bending. She also noticed decreased strength in addition to tenderness, swelling, and crepitus during movement. The wrist range of motion (ROM) was 0‐20° flexion and extension, and 10° lateral deviation ulnar or radially. An informed consent was obtained after risks, and benefits were reviewed to receive the treatment via intra‐articular injection of AMUC particulate (Clarix FLO^®^; Amniox). In brief, the left wrist was prepped with povidone‐iodine (Betadine; Purdue Pharma) and vapocoolant (Pain Ease, Gebauer Company) followed by injection of 2 cc of 1% lidocaine (Hospira) using a 30G needle. The 100 mg of AMUC particulate was reconstituted with 2 cc of preservative‐free normal saline and injected in an equal volume into the scapholunate joint and mid‐carpal joint using a 25G needle under ultrasound guidance (LOGIQ e, GE Healthcare) using a 12 MHz linear probe. No complications were observed within 30 minutes, and the patient was released home with the instruction to wear a neutral carpal tunnel splint for 6 weeks.

Within 3‐5 days after injection, the patient noted similar pain while on acetaminophen (Tylenol). However, at one month post‐injection, the patient reported no pain with and without (during showers) the splint aside from “an occasional ache” as described by the patient. Physical examination revealed decreased swelling, crepitus, and tenderness. The ROM had also improved to 30 degrees of flexion and extension without pain. At 3 months post‐injection, the patient reported no sharp pains with occasional dull aches that had severity of 2‐3 out of 10. Patient also discontinued use of the splint 2 weeks prior to the 3‐month visit and subsequently reported weakness in her hand and arm due to deconditioning. Overall, the patient reported her overall patient global impression of change as 70% improved compared to pre‐injection (on a scale from 0% to 100%, with 0% the same as before injection and 100% was completely normal).^14^ No complications or adverse events were encountered.

## 3 DISCUSSION

Herein, we present the successful treatment by intra‐articular injection of AMUC particulate to relieve severe, chronic (>1 year) wrist OA pain secondary to SLAC that was refractory to conservative medical treatments. Considering there is no gold standard nonsurgical treatment and many elderly patients decline surgical intervention, injection of AMUC particulate may be an alternative to manage such refractory wrist OA pain.

The clinical benefit might arise from AMUCs anti‐inflammatory and antiscarring actions.^7^, ^8^, ^15^, ^16^ In particular, AMUC's innate, major biochemical component, that is, HC‐HA/PTX3, has been shown to be predominantly responsible for its therapeutic effects^7^ by promoting apoptosis of activated neutrophils^17^, ^18^ and promoting M2 macrophage polarization.^18^ Furthermore, AMUC tissues contain IL‐1 receptor antagonist (IL‐1RA) and IL‐10, which suppress inflammation mediated by IL‐1 and IL‐6, respectively.^15^, ^19^, ^20^ IL‐1 and IL‐6 are main inflammatory and catabolic cytokines in the pathophysiology of OA, and elevated levels are highly predictive of OA severity.^21^, ^22^, ^23^, ^24^, ^25^, ^26^, ^27^

## 4 CONCLUSION

This case report highlights the utilization of amniotic and umbilical cord particulate to alleviate moderate to severe pain in a patient suffering from wrist osteoarthritis associated with scapholunate dissociation, scapholunate ligament tear, and avascular necrosis of the lunate.

## CONFLICT OF INTEREST

No benefits in any form have been received or will be received from a commercial party related directly or indirectly to the subject of this article.

## AUTHOR CONTRIBUTION

SK: contributed to this work.
